# The Efficacy of Cheonggukjang in Alleviating Menopausal Syndrome and Its Effects on the Gut Microbiome: A Randomized, Double-Blind Trial

**DOI:** 10.3390/nu17030505

**Published:** 2025-01-30

**Authors:** A-Lum Han, Myeong-Seon Ryu, Hee-Jong Yang, Do-Youn Jeong, Keum-Ha Choi

**Affiliations:** 1 Department of Family Medicine, Wonkwang University Hospital, Iksan 54538, Republic of Korea; 2 Microbial Institute for Fermentation Industry, Sunchang 56048, Republic of Korea; rms6223@naver.com (M.-S.R.); yo217@naver.com (H.-J.Y.); godfiltss@naver.com (D.-Y.J.); 3 Departments of Pathology, Wonkwang University Hospital, Iksan 54538, Republic of Korea; jdy2534@korea.kr

**Keywords:** menopausal syndrome, Cheonggukjang, gut microbiome, metabolic health, Kupperman index

## Abstract

**Background/Objectives**: Menopause is associated with various symptoms. Although hormone replacement therapy (HRT) is commonly used, concerns regarding its side effects have led to the development of alternative treatments. This study evaluated the potential health benefits of Cheonggukjang, a traditional Korean fermented soybean product in alleviating menopausal symptoms and improving metabolic parameters in postmenopausal women. Additionally, the effect of Cheonggukjang on the gut microbiome was assessed using stool analysis. **Methods**: In this randomized, double-blind clinical trial, 60 postmenopausal women were assigned to three groups: high-beneficial-microorganism content Cheonggukjang (HTC), low-beneficial-microorganism content Cheonggukjang (LTC), and commercially available Cheonggukjang (CC). Participants consumed 3.3 g of Cheonggukjang tablets daily for 8 weeks. We assessed menopausal symptom relief using the Kupperman index, metabolic parameters, and changes in the gut microbiome using stool analysis. **Results**: The Kupperman index scores significantly decreased across all three groups, with the HTC group showing the greatest improvement. No significant changes were observed in body mass index, weight, or lipid profiles. Blood glucose levels decreased significantly only in the HTC group. Microbiome analysis revealed an increase in beneficial bacteria in the HTC and CC groups and a decrease in harmful bacteria. The Firmicutes-to-Bacteroidetes ratio decreased in both HTC and CC groups, though this change was not significant. **Conclusions**: Cheonggukjang supplementation significantly alleviated menopausal symptoms, particularly in the HTC group, and improved the gut microbiota composition. These findings suggest that Cheonggukjang, particularly with its high beneficial microorganism content, may offer a promising alternative for managing menopausal symptoms and improving metabolic health in postmenopausal women.

## 1. Introduction

Menopause is a phase in a woman’s life characterized by the cessation of reproductive function, marking the transition from fertility to the non-reproductive stage. This period is associated with a gradual decline in ovarian function over several years, which leads to significant alterations in hormone levels and induces various physical and psychological changes. These changes often manifest as vasomotor symptoms, including hot flashes and sweating, as well as sleep disturbances, urogenital atrophy, irritability, emotional instability, and heightened anxiety. Moreover, the incidence of conditions such as osteoporosis and cardiovascular diseases tends to increase. The severity and presentation of these symptoms can vary considerably among individuals and are often classified as part of the menopausal syndrome [1].

Estrogen has been utilized in hormone replacement therapy (HRT) for almost six decades to manage menopausal symptoms, including disorders related to glucose metabolism. Following the release of the Women’s Health Initiative Study in 2002, the scrutiny of HRT use has grown [2]. While HRT has been associated with an increased risk of breast cancer and cardiovascular disease, evidence remains contentious. Despite its potential adverse effects, HRT has demonstrated positive effects on various metabolic parameters, such as improving insulin resistance [3]. Concerns regarding the side effects of estrogen have prompted individuals to seek alternative treatments to manage menopausal symptoms. As a result, a growing number of menopausal women are turning to herbal remedies and dietary supplements to enhance their quality of life [4].

The ideal HRT would replicate the positive effects of estrogen while avoiding harmful side effects. As researchers seek safer and more tolerable alternatives to HRT in postmenopausal women, natural selective estrogen receptor modulators (SERMs) have gained considerable attention. Epidemiological studies have shown that American women experience significantly higher mortality from coronary heart disease than their Japanese counterparts. Notably, among Asian immigrants, those who retained their traditional diet had lower coronary heart disease rates than those who adopted a Westernized diet; this disparity is mainly attributed to the high intake of soy foods in Asian diets [5].

Isoflavones, the active compounds in soy, have a structure similar to that of endogenous estrogen, bind to estrogen receptors, and function as natural SERMs. Studies suggest that isoflavones may lower the risk of certain cancers, including lung, prostate, colon (in women only), and breast cancers, without any associated risk of thrombosis or stroke [6]. In addition, isoflavones improve metabolic parameters in postmenopausal women. Previous research has shown that isoflavones can positively impact lipid profiles by reducing serum total cholesterol (TC), low-density lipoprotein cholesterol (LDL-C), and triglycerides (TGs) while increasing high-density lipoprotein cholesterol (HDL-C) [7].

The biological activities of isoflavones can result in both prolonged genomic effects, mediated through intracellular estrogen receptors, and immediate non-genomic effects, which involve various intracellular signaling pathways. Clinical investigations have indicated that phytoestrogens may enhance glucose metabolism, as observed in several studies on menopausal women [8]. Recent studies have shown that extracts from certain plants can aid in metabolic management, particularly in individuals with impaired fasting glycemia [9].

The intake of fermented foods, in addition to fermented soy products, can influence the gut microbiome and its metabolites [10]. A study involving 115 participants who consumed fermented foods at varying frequencies over a 4-week period revealed that their microbial communities included microbes typically found in fermented foods (such as *Lactobacillus acidophilus*, *Levilactobacillus brevis*, *Lactobacillus kefiranofaciens*, *Lentilactobacillus parabuchneri*, *Lactobacillus helveticus*, and *Latilactobacillus sakei*), as well as microbes not commonly associated with fermented products. The latter group includes species such as *Streptococcus dysgalactiae*, *Prevotella melaninogenica*, *Enorma massiliensis*, *Prevotella multiformis*, *Enterococcus cecorum*, and *Bacteroides paurosaccharolyticus* [11].

In this study, we investigated Cheonggukjang, a fermented soybean product commonly consumed in Asian diets, in a clinical trial to explore its health benefits in women with menopausal syndrome. Cheonggukjang is a traditional Korean food, rich in essential nutrients such as amino, fatty, and organic acids, minerals, and vitamins, making it an important functional food [12]. This study aimed to evaluate the potential of Cheonggukjang to mitigate menopausal symptoms and improve metabolic parameters in postmenopausal women. In addition, this study sought to compare the effectiveness of traditional Cheonggukjang with that of its commercial counterpart. Furthermore, we examined whether a higher concentration of beneficial microbes in Cheonggukjang could amplify its therapeutic effects. To the best of our knowledge, no clinical investigations have focused on the influence of the Cheonggukjang production method on alleviating menopausal symptoms. This study is noteworthy as it not only compared different Cheonggukjang production methods but also assessed changes in the fecal microbiome after Cheonggukjang consumption.

## 2. Materials and Methods

### 2.1. Production of Experimental Cheonggukjang Tablets

In the traditional preparation of Cheonggukjang, rice straw served as the inoculum for fermentation. The soybeans were soaked and cooked until they became tender. Once softened, the beans were covered with rice straw and placed in a warm, humid environment, typically maintaining a temperature range of 35–40 °C and a humidity of approximately 80%. This environment promotes the growth of microorganisms during fermentation, which lasts between 24 and 72 h.

**Figure 1:**
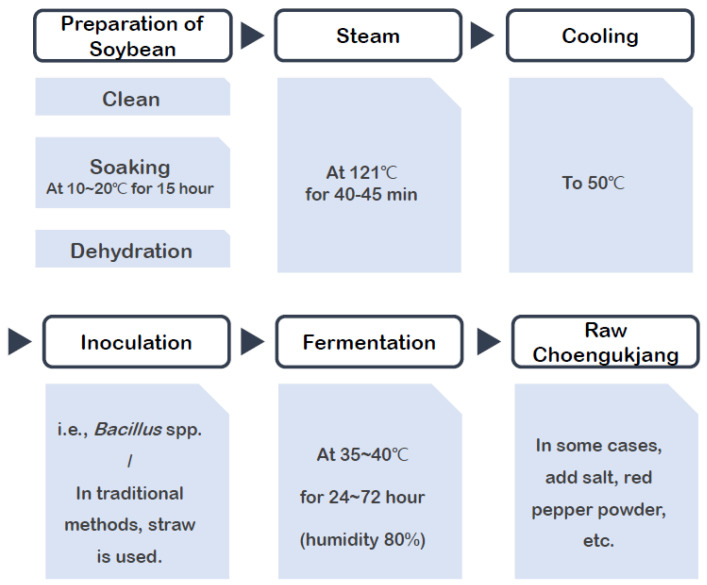
Traditional Cheonggukjang manufacturing process.

The process of making traditional Cheonggukjang is depicted in Figure 1. For clinical trial purposes, Cheonggukjang was freeze-dried and ground into a powder. This powder was then mixed with excipients in accordance with the ratios detailed in Table 1 before tablet production.

High-beneficial-microorganism content (HTC) and low-beneficial-microorganism content (LTC) Cheonggukjang products were prepared using traditional manufacturing and management processes. Microbial community analysis was conducted to investigate the microbial distribution generated during the natural fermentation process. Based on the results, the products were classified into two categories based on the differences in the content of beneficial microorganisms (microorganisms permitted for use in food materials by the Ministry of Food and Drug Safety): high content (41.49% beneficial microorganisms) and low content (37.29% beneficial microorganisms). The commercially available Cheonggukjang (CC) was produced through an artificial fermentation process using a specific microbial strain and was used as a factory-produced control group for comparison with the traditional Cheonggukjang products.

### 2.2. Study Design

This study was designed as a randomized, double-blind clinical trial conducted over an 8-week period (Registration Number KCT 0010017). The participants visited the research center three times during the course of the trial. After 8 weeks of Cheonggukjang supplementation, participants returned for evaluation of vital signs, adverse effects, and medication adherence. On both the initial and final study days, we assessed markers related to menopausal symptom relief, including the Kupperman index. In addition, we evaluated efficacy indicators, such as weight, bioelectrical impedance analysis results, inflammatory markers, lipid profiles, and metabolic markers related to glucose metabolism, including insulin resistance and blood glucose levels.

Participants were assigned to groups using block randomization (1:1:1 ratio) with fixed block sizes (e.g., 4, 6, and 8). Randomization was performed by an independent statistician, and both participants and researchers were blinded. Unblinding occurred only in emergencies, with proper documentation maintained.

The participants were assigned to receive three different types of Cheonggukjang tablets: Cheonggukjang with HTC, Cheonggukjang with LTC, and CC. Microbiome analysis, conducted in accordance with verification by the Korean Ministry of Food and Drug Safety, confirmed that a product with a high microorganism content was classified as HTC, whereas that with a lower microorganism content was classified as LTC [13].

Of the 62 participants initially enrolled, 2 withdrew, leaving 60 participants who completed the clinical trial. Each participant was assigned a screening number upon providing written informed consent, with numbers ranging from 01 to 62, which also served as their identification code. Throughout the study, participants were instructed to refrain from using medications or dietary supplements other than the assigned Cheonggukjang tablets and to maintain their usual diet and activity levels.

### 2.3. Participants

Volunteers with a body mass index (BMI) ≥ 23 kg/m^2^, aged between 19 and 70 years, were recruited and randomly allocated to three groups. The exclusion criteria were a change in body weight of >10% over the preceding 3 months; cardiovascular conditions such as arrhythmias, heart failure, myocardial infarction, or the use of a pacemaker; allergies or hypersensitivity to any component of the test product; gastrointestinal diseases, including Crohn’s disease; a history of gastrointestinal surgeries (e.g., appendectomy or bowel surgery); participation in another clinical trial within the past 2 months; liver dysfunction or acute/chronic kidney disease; use of antipsychotic medications within the past 2 months; abnormal laboratory findings as assessed by the investigator; psychological disorders; a history of alcohol or drug abuse; and pregnancy or breastfeeding. The study protocol was approved by the Institutional Review Board of Wonkwang University Hospital (IRB No.: WKUH 2024-04-019).

Participants were instructed to maintain a dietary diary and record all food intake as accurately as possible. Upon visiting the center, participants completed a physical activity questionnaire based on the Global Physical Activity Questionnaire [14]. Each participant consumed 3.3 g of Cheonggukjang tablets (equivalent to 3.3 g of fermented soybeans) daily.

### 2.4. Assessment of Improvement in Menopausal Syndrome

The Kupperman index, developed by Kupperman et al., is a widely used diagnostic tool for menopausal syndrome, both globally and in Asia [13]. It categorizes menopausal symptoms into six main groups: vasomotor symptoms, urinary tract issues, psychoneurological and motor symptoms, digestive disturbances, and systemic symptoms [15]. The index comprises 25 questions that evaluate these six categories, helping to assess the severity and characteristics of the symptoms. The total score is interpreted as follows: a score of 20 or less indicates mild symptoms, 20–40 points suggest moderate symptoms, and scores between 40 and 60 represent severe symptoms. Scores > 60 are considered indicative of very severe menopausal syndrome.

### 2.5. Safety Evaluation

Blood chemistry and hematologic tests were performed to assess the safety of the intervention and to monitor liver and kidney function. The following parameters were assessed: white and red blood cell count, hemoglobin, hematocrit, platelet count, total protein, albumin, alanine aminotransferase (ALT), aspartate aminotransferase (AST), blood urea nitrogen (BUN), and creatinine. Blood pressure and pulse were measured after a 10-min rest at each visit. The participants were also instructed to report any adverse symptoms or side effects experienced during the course of Cheonggukjang supplementation.

### 2.6. Metabolic Parameter Assessment

The participants fasted for >12 h before blood sample collection. The following metabolic parameters were analyzed using a Hitachi 7600 automatic analyzer (Hitachi, Tokyo, Japan): TC, LDL-C, HDL-C, ALT, AST, gamma-glutamyl transferase (GGT), BUN, creatinine, glucose, insulin, and high-sensitivity C-reactive protein (hs-CRP). Additionally, insulin resistance was evaluated using the Homeostasis Model Assessment of Insulin Resistance (HOMA-IR), insulin secretion with HOMA-beta (HOMA-β), and the Quantitative Insulin Sensitivity Check Index (QUICKI).

### 2.7. Gut Microbiome Analysis

To assess the impact of the intervention on the gut microbiome, participants provided stool samples at baseline and again after 8 weeks. Using the MICROBE and ME Stool Collection Kits (Macrogen, Seoul, Republic of Korea), participants collected at least 1 g of feces, which were then stored in a frozen state for subsequent microbiome analysis.

Fecal samples were analyzed using next-generation sequencing (NGS) techniques. Bacterial DNA was extracted from the fecal samples using the Power Water DNA Isolation Kit (Qiagen, Valencia, CA, USA) following the manufacturer’s instructions. The 16S rRNA gene was then amplified using PCR with universal 16S primers, and the resulting amplicons were used to prepare sequencing libraries according to standard protocols. Sequencing was conducted on the Illumina MiSeq platform, and sequence data were processed using Mothur v.1.36 to determine the taxonomy and relative abundance of bacterial species in each sample. A detailed taxonomic analysis was performed, including classification at the order level, and principal coordinate analysis (PCoA) was conducted to visualize the relationships between bacterial communities across samples.

### 2.8. Statistical Analysis

All statistical analyses were conducted using SPSS version 23.0 (IBM Corp., Armonk, NY, USA). The results are expressed as mean ± standard error or as percentages for categorical data. A *p*-value of less than 0.05 was considered indicative of statistical significance.

The sample size was calculated to ensure 80% power at an alpha level of 0.05, with a dropout rate of 20%. Efficacy parameters were evaluated in the per-protocol group, whereas safety data were analyzed using the intention-to-treat approach. Baseline differences in categorical variables between the groups were assessed using the chi-square test. To examine the changes before and after the 8-week intervention, paired *t*-tests were performed. A linear mixed-effects model was used to analyze continuous outcome variables with repeated measures. Furthermore, 24 h dietary intake data were processed using Can-Pro 3.0 software (Korean Nutrition Society, Seoul, Republic of Korea).

## 3. Results

### 3.1. Baseline Participant Information

**Figure 2:**
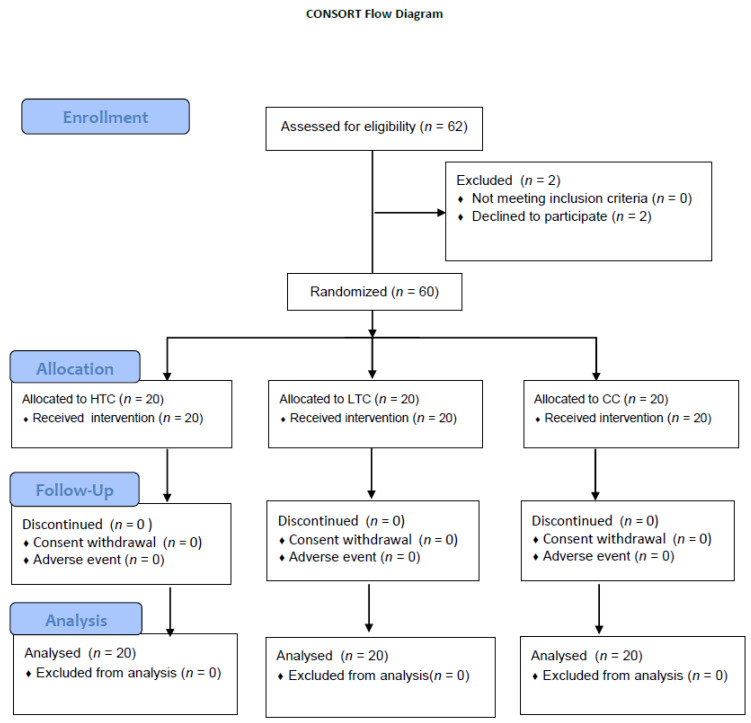
CONSORT flow diagram.

Sixty participants were included in the final analysis: 20 in the HTC group, 20 in the LTC group, and 20 in the CC group (Figure 2). No adverse events were reported by any of the participants following the completion of the study.

### 3.2. Anthropometric Parameters

Table 2 shows the baseline characteristics of the participants. A cross-sectional analysis was conducted to examine variables such as alcohol consumption and smoking habits, while a one-way analysis of variance was used to assess differences in age, weight, and BMI. No significant differences were observed among the three groups in terms of age, weight, height, alcohol consumption, smoking status, baseline weight, or BMI. Dietary intake assessments showed no notable changes in caloric consumption, either within or between the groups. Additionally, the physical activity questionnaire revealed no significant differences in the metabolic equivalents of the tasks across or between the groups. Data on age at menarche, age at menopause, the duration of menopause, and the number of childbirths were collected from all participants, and no significant differences were found between the groups (Table 3).

### 3.3. Safety Evaluation

Blood tests assessing liver function, kidney health, and general hematologic parameters showed normal levels after the administration of Cheonggukjang pills (Table 4). Notably, in the HTC group, AST and ALT levels decreased after Cheonggukjang consumption. No adverse symptoms or unusual effects were reported by the participants in any of the groups after taking Cheonggukjang tablets.

### 3.4. Effect on Metabolic Parameters

After the administration of Cheonggukjang tablets, blood glucose levels decreased only in the CC group. However, no significant changes were observed in insulin, HOMA-IR, HOMA-β, or QUICKI levels in any group. Additionally, the lipid profiles across all three groups did not differ significantly. Moreover, Cheonggukjang showed no significant anti-obesity effects (Table 5).

### 3.5. Efficacy Evaluation of Kupperman Index Scores Across the Three Groups

**Figure 3:**
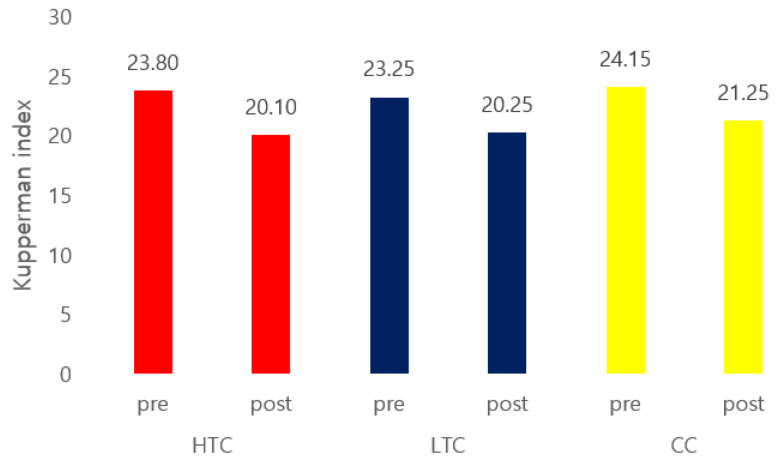
Efficacy evaluation of the Kupperman index scores across the three groups. Note: HTC, traditional Cheonggukjang containing a high dose of beneficial microbes; LTC, traditional Cheonggukjang containing a low dose of effective microbes; CC, commercially prepared Cheonggukjang.

Following Cheonggukjang tablet administration, significant improvements were observed in the Kupperman index scores, with a marked reduction in the total scores across all three groups. In addition to the overall score, changes in individual symptom scores were assessed (Figure 3). A reduction in insomnia was noted in all groups. Hot flashes improved in both the HTC and LTC groups, whereas paresthesia and nervousness symptoms were reduced in the HTC and CC groups. Notably, the HTC group exhibited improvements in four distinct symptoms, showing the highest number of improvements across the three groups (Table 6).

### 3.6. Analysis of the Fecal Gut Microbiome

**Figure 4:**
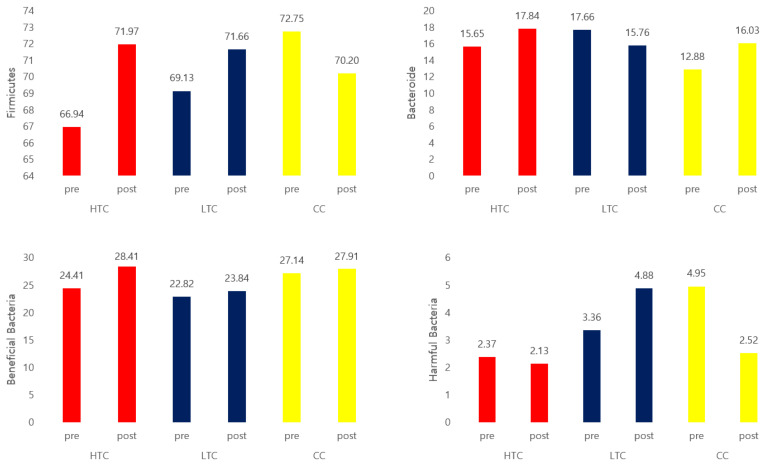
Microbiome analysis of feces. Note: HTC, traditional Cheonggukjang containing a high dose of beneficial microbes; LTC, traditional Cheonggukjang containing a low dose of effective microbes; CC, commercially prepared Cheonggukjang.

The abundance of Firmicutes decreased, whereas that of Bacteroidetes increased in the HTC and CC groups, although these changes were not statistically significant (Figure 4). The Firmicutes-to-Bacteroidetes (F/B) ratio decreased in the HTC and CC groups. However, these changes were not statistically significant (Table 7).

Cheonggukjang supplementation increased the population of beneficial bacteria exclusively in the HTC group, whereas no decrease in harmful bacteria was noted in any of the three groups (Table 7). The beneficial microorganisms identified included *Lactobacillus* spp., *Bifidobacterium* spp., *Lactococcus lactis, Enterococcus faecium*, and *Bacteroides* spp. The harmful bacteria detected included *Clostridium perfringens, Bacteroides eggerthii, Sutterella stercoricanis, Ruminococcus torques, Parabacteroides merdae*, and *Parabacteroides distasonis* (Table 8).

## 4. Discussion

This study aimed to compare the effectiveness of traditional and commercial Cheonggukjang in alleviating menopausal symptoms. Additionally, we examined whether traditional Cheonggukjang would have beneficial effects if it contained high concentrations of beneficial microbes. Cheonggukjang is a traditional Korean food made from fermented boiled soybeans and rice straw. It is considered a functional food due to its content of various biologically active compounds, including isoflavones, phytic acid, saponins, trypsin inhibitors, tocopherols, unsaturated fatty acids, dietary fiber, oligosaccharides, antioxidants, and thrombolytic enzymes.

Cheonggukjang was chosen for its rapid fermentation and higher concentration of beneficial microorganisms, which sets it apart from other fermented soy products such as miso, tempeh, and natto [16,17]. Cheonggukjang’s probiotic-rich content makes it particularly suitable for exploring its effects on gut microbiota and metabolic health in postmenopausal women [16,17].

Given its traditional use in Korea for digestive and immune health, Cheonggukjang offers a promising approach to managing menopausal symptoms. Future studies could compare its effects with other fermented soy products to better understand the role of fermentation and microbial profiles in health benefits.

Cheonggukjang is categorized into traditional and commercial forms based on its production method. Traditionally produced Cheonggukjang is fermented at 40–43 °C for 2–3 days using natural microbial flora, including *Bacillus subtilis*, which leads to variability in the final product and a distinct fermentation odor. This method results in a greater inconsistency in quality [16].

On the other hand, commercially produced Cheonggukjang uses controlled fermentation with selected *B. subtilis* strains, ensuring uniformity and consistency. The process may also include modifications to reduce the strong fermentation odor, making the product more appealing to global markets. The primary differences are in microbial control, fermentation conditions, and product consistency [17].

Traditional Cheonggukjang can be classified into two categories based on its microbiome composition: high-effective microorganisms and low-effective microorganisms. Participants who consumed each form of Cheonggukjang were further divided into three groups, and the differences in the effects between these groups were evaluated. The alleviation of menopausal symptoms in the three groups was evaluated using the Kupperman index to assess the degree of improvement following Cheonggukjang tablet administration. Changes in lipid profiles, glucose levels, and insulin resistance markers were assessed following Cheonggukjang consumption.

The findings of this study revealed a significant reduction in the mean Kupperman index following Cheonggukjang supplementation, with significant differences observed among the three groups. The HTC group exhibited the largest decrease in the total Kupperman index score. The HTC group showed a significant decrease in the scores for the three Kupperman index items, demonstrating the greatest number of improvements among the groups. The degree of improvement for each symptom varied across the groups, with no symptoms showing an increase in the score.

Cheonggukjang supplementation reduced blood glucose levels across all three groups, although no significant changes were observed in insulin resistance markers. Additionally, the number of beneficial bacteria increased exclusively in the HTC group after supplementation.

The early stages of menopause are often associated with an increased risk of metabolic syndrome, hot flashes, vaginal dryness, sleep disturbances, and other symptoms. Estrogen therapy, with or without progesterone, effectively prevents many of these changes [18]. However, due to the findings of the Women’s Health Initiative, which suggested that the overall risks of estrogen therapy may outweigh its benefits, an increasing number of menopausal women are choosing to forgo estrogen therapy and are seeking alternative treatments [19]. Soy-derived products have been proposed as promising alternatives, potentially offering benefits similar to estrogen, without associated risks [20].

In this study, we selected Cheonggukjang tablets, a soybean-based product commonly consumed in Korea. Soybeans are an excellent source of plant-based proteins and calcium and are rich in isoflavones, including daidzein, genistein, and glycitein [21]. Typically, soybeans contain 0.1–5 mg of total isoflavones per gram, primarily in their glycosylated forms [22]. However, during the fermentation process used to produce Cheonggukjang, the concentration of aglycone isoflavones increases 10- to 100-fold, thereby enhancing their bioavailability and potential health benefits [22].

Cheonggukjang fermentation involves microorganisms, particularly strains of the *Bacillus* genus [23]. During fermentation, *Bacillus* species break down proteins and other nutrients in soybeans, resulting in the production of various compounds, including sticky, mucus-like substances. This mucus-like substance is a complex mixture containing compounds such as levan and polyglutamate. Additionally, the health effects of Cheonggukjang vary depending on whether it is fermented using traditional methods or produced in commercial settings [23]. Even with traditional methods, health benefits differ based on the concentration of beneficial microbial strains.

In our study, the total Kupperman index decreased after Cheonggukjang tablet administration in all three groups, indicating an improvement in menopausal syndrome symptoms. In a study similar to ours, significant reductions in the Kupperman index were observed following treatment with isoflavones. Sixty postmenopausal women were randomized to receive either red clover (*Trifolium pratense*) isoflavones or a placebo for 12 weeks. Both treatments significantly reduced the Kupperman index, with a greater decrease following isoflavone administration (baseline: 27.2 ± 7.7; after isoflavones: 5.9 ± 3.9; after placebo: 20.9 ± 5.3, *p* < 0.05) [24].

When analyzing each item of the Kupperman index separately, improvements in hot flashes, as measured by the Kupperman Menopause Index, were observed only in the HTC and LTC groups. A systematic review and meta-analysis revealed that the consumption of isoflavones, whether derived from soy or synthetically produced to replicate those found in soy, resulted in a more significant reduction in hot flashes than that following placebo consumption [25].

A similar result to that of our study was observed in a study that administered a daily dose of 40–60 mg of daidzein in daidzein-rich isoflavone aglycones [26]. A review article reported that soybean consumption resulted in a 45% reduction in hot flash frequency, whereas the placebo group showed a 30% decrease [27]. Furthermore, a separate study found that daily supplementation with 54 mg of the phytoestrogen genistein could reduce the frequency and severity of hot flashes over 1 to 2 years in postmenopausal women [28]. This suggests that both daidzein and genistein, derived from soy isoflavones, can alleviate postmenopausal symptoms. However, some studies have reported no significant effect of soy isoflavones on these symptoms in White women [29].

When analyzing each item of the Kupperman index separately, improvements in insomnia, as measured by the Kupperman Menopause Index, were not observed in any of the three groups. In contrast to our findings, a randomized controlled trial conducted on postmenopausal women with insomnia found a significant improvement in sleep efficiency in the isoflavone treatment group compared to that in the placebo group [30]. A prior study involving 169 postmenopausal women demonstrated that isoflavone treatment effectively enhanced the quality of life and alleviated climacteric symptoms, including sleep disturbances [31].

During menopause, the ability to metabolize glucose gradually declines, leading to an increased prevalence of diabetes and impaired glucose tolerance. Lower-dose isoflavone supplementation is more effective than higher-dose supplementation in reducing blood glucose levels [32]. Although several studies have shown no significant reduction in blood glucose levels after short-term supplementation with soy isoflavones [33], the current study observed decreased blood glucose levels across all three groups following Cheonggukjang supplementation. However, no significant changes were observed in insulin resistance markers.

In our study, no improvement in the lipid profile was observed in any of the three groups following Cheonggukjang tablet administration. A meta-analysis evaluating the impact of isoflavone-containing soybean extracts on dyslipidemia revealed significant reductions in LDL-C, TC, and TG levels, along with a notable increase in HDL-C levels [34].

This study investigated gut-related outcomes; therefore, the co-administration of certain antibiotics and dietary supplements that could affect the results was avoided. Specifically, participants were instructed to refrain from using broad-spectrum antibiotics such as penicillins, cephalosporins, and quinolones, as well as gut-related dietary supplements, including probiotics, prebiotics (e.g., inulin and fructooligosaccharides), and high-fiber substances (e.g., guar gum and glucomannan). The numbers of Firmicutes decreased and those of Bacteroidetes increased in the HTC and CC groups, although the changes were not statistically significant. The F/B ratio decreased in the HTC and CC groups; however, this difference was not statistically significant. Following supplementation, an increase in the population of beneficial bacteria was observed exclusively in the HTC group, whereas no decrease in the population of harmful bacteria was observed in any of the three groups.

The gut microbiota is a diverse ecosystem comprising all bacterial species permanently inhabiting the gastrointestinal tract, along with various microorganisms originating from the environment. This microbiota is essential for supporting the physiological functions of the host. In healthy adult humans, the gut microbiota predominantly comprises two main phyla: Firmicutes and Bacteroidetes [35]. The F/B ratio is an important indicator for evaluating the composition of the gut microbiota. Studies have demonstrated that this ratio is linked to body composition, particularly BMI. In particular, obese individuals typically exhibit a higher proportion of Firmicutes and a reduced abundance of Bacteroidetes in their gut microbiota [36].

Several studies have observed a positive correlation between the decrease in F/B ratio and both fasting and postprandial blood glucose levels, although this correlation was not significant [37,38]. A study investigating the effects of fresh and fermented kimchi on patients with obesity found that both types of kimchi induced changes in the gut microbiota. Notably, fermented kimchi increased Actinobacteria, which is negatively correlated with body fat, as well as Bacteroides and Prevotella, whereas Blautia decreased [39]. Fermented foods can alter the gut microbiome and provide various health benefits. The isoflavones and their metabolites found in various Asian fermented foods enhance these effects, promoting positive changes in the gut environment and potentially contributing to the prevention and management of various diseases [40].

This study had several limitations. First, although the participants were instructed not to alter their dietary habits, food preferences, physical activity, or exercise routines, we could not adequately control for these factors. Second, due to the short duration of the study, we were unable to administer Cheonggukjang tablets over an extended period. Third, the small sample size limits the generalizability of the results. Additionally, since the primary objective was to assess the effectiveness of traditional Cheonggukjang, no control group was included, which may have introduced bias due to the placebo effect.

## 5. Conclusions

This study demonstrated that Cheonggukjang supplementation significantly alleviated menopausal symptoms, with the most pronounced effects observed in the HTC group, which contained higher concentrations of beneficial microorganisms. Traditional Cheonggukjang, particularly when enriched with microbial content, showed enhanced efficacy in reducing Kupperman index scores, improving specific symptoms such as hot flashes and glucose levels, and increasing the population of beneficial gut bacteria. The results of this study demonstrate the safety and stability of Cheonggukjang tablets at the 3.3 g dosage. This finding aligns with the prior safety assurance provided by the Ministry of Food and Drug Safety.

Although these findings support the potential benefits of traditional Cheonggukjang as a functional food for menopausal women, certain limitations warrant further investigation. The study’s short duration, small sample size, and absence of a control group may have influenced the results. Additionally, although blood glucose levels improved, no significant changes were observed in lipid profiles or insulin resistance markers, highlighting the need for more comprehensive long-term studies to confirm these outcomes.

Future studies should explore the effects of Cheonggukjang over extended periods of time, incorporate larger and more diverse populations, and include controlled dietary and lifestyle monitoring. Investigating the mechanisms underlying the interactions between Cheonggukjang-derived bioactive compounds and the gut microbiota could further elucidate their role in managing menopausal symptoms and metabolic health. Future studies should explore personalized Cheonggukjang-based intervention strategies for menopausal symptom management based on individual microbiota profiles or genetic predispositions. This study underscores the importance of traditional fermentation methods and microbiome diversity in maximizing the therapeutic potential of fermented foods.

## Figures and Tables

**Table 1:** Composition of the Cheonggukjang tablets.

	HTC		LTC		CC	
	Content (g)	Ratio (%)	Content (g)	Ratio (%)	Content (g)	Ratio (%)
Freeze-dried Cheonggukjang powder	2.97	90	2.97	90	2.97	90
Glutinous rice flour	0.33	10	0.33	10	0.33	10
Total	3.3	100	3.3	100	3.3	100

HTC, traditional Cheonggukjang containing a high dose of beneficial microbes; LTC, traditional Cheonggukjang containing a low dose of effective microbes; CC, commercially prepared Cheonggukjang.

**Table 2:** Anthropometric parameters of the participants.

Value	Group			
	HTC (*n* = 20)	LTC (*n* = 20)	CC (*n* = 20)	*p*-Value
Drinking (*n*)	20 (100)	20 (100)	20 (100)	-
Smoking (*n*)	20 (100)	20(100)	20 (100)	-
Age (years)	58.20 ± 3.98	57.70 ± 4.95	59.05 ± 5.02	0.655
Weight (kg)	66.59 ± 6.89	65.80 ± 7.46	65.36 ± 7.92	0.870
BMI (kg m⁻^2^)	26.78 ± 2.53	26.48 ± 2.99	26.70 ± 2.55	0.936

Note: HTC, traditional Cheonggukjang containing a high dose of beneficial microbes; LTC, traditional Cheonggukjang containing a low dose of effective microbes; CC, commercially prepared Cheonggukjang; BMI, body mass index. Values are presented as mean ± standard deviation or number (percentage).

**Table 3:** Menopause-related factors.

Value	Group			
	HTC (*n* = 20)	LTC (*n* = 20)	CC (*n* = 20)	*p*-Value
Menarche age (years)	14.80 ± 1.28	14.95 ± 1.47	15.55 ± 1.43	0.208
Menopause age (years)	51.85 ± 2.01	51.20 ± 3.78	52.00 ± 3.13	0.681
Menopause period (years)	77.80 ± 51.68	79.40 ± 74.24	85.60 ± 62.87	0.920
Number of births	2.25 ± 0.85	2.25 ± 0.64	2.20 ± 0.70	0.970

Note: HTC, traditional Cheonggukjang containing a high dose of beneficial microbes; LTC, traditional Cheonggukjang containing a low dose of effective microbes; CC, commercially prepared Cheonggukjang. Values are presented as mean ± standard deviation or number (percentage).

**Table 4:** Safety measurements.

Value	Group								
	HTC (*n* = 20)			LTC (*n* = 20)			CC (*n* = 20)		
	Before	After	*p*-Value	Before	After	*p*-Value	Before	After	*p*-Value
WBC (10^3^/µL)	5.93 ± 1.16	5.89 ± 1.19	0.802	6 ± 1.82	5.88 ± 1.28	0.745	6.84 ± 1.59	6.89 ± 2.09	0.892
RBC (10^6^/µL)	4.51 ± 0.22	4.44 ± 0.25	0.044	4.43 ± 0.2	4.38 ± 0.29	0.283	4.43 ± 0.25	4.4 ± 0.34	0.428
Hemoglobin (g/dL)	13.84 ± 0.74	13.69 ± 0.75	0.153	13.49 ± 0.62	13.48 ± 0.87	0.971	13.41 ± 0.64	13.42 ± 0.88	0.967
Hematocrit (%)	40.96 ± 2.34	39.93 ± 2	0.010	40.29 ± 1.55	39.7 ± 2.54	0.141	40.04 ± 1.84	39.55 ± 2.38	0.170
GGT (IU/L)	30.6 ± 20.91	26.85 ± 14.06	0.056	29.5 ± 22.21	33.65 ± 19.91	0.141	29.45 ± 23.02	26.95 ± 14.85	0.245
AST (IU/L)	30.5 ± 10.44	26.35 ± 8.32	0.011	24.35 ± 5.5	25 ± 7.28	0.557	24.6 ± 5.28	24.95 ± 5.91	0.760
ALT (IU/L)	30.05 ± 14.73	25 ± 9.86	0.003	26.2 ± 6.9	26.7 ± 11.85	0.755	25.5 ± 8.43	24.15 ± 8.29	0.207
BUN (mg/dL)	14.32 ± 2.82	13.5 ± 2.65	0.238	14.25 ± 3.03	14.72 ± 2.85	0.580	15.45 ± 3.29	15.8 ± 3.98	0.731
Creatinine (mg/dL)	0.67 ± 0.08	0.65 ± 0.11	0.297	0.7 ± 0.14	0.66 ± 0.14	0.088	0.65 ± 0.11	0.63 ± 0.15	0.494
Uric acid (mg/dL)	4.63 ± 1.26	4.67 ± 1.05	0.804	4.64 ± 1.07	4.75 ± 1.22	0.370	4.59 ± 0.93	5.01 ± 1.28	0.032
T-protein (g/dL)	6.85 ± 0.35	6.9 ± 0.37	0.419	6.96 ± 0.43	7.03 ± 0.45	0.213	6.78 ± 0.36	6.93 ± 0.44	0.102
Albumin (g/dL)	4.26 ± 0.18	4.27 ± 0.15	0.871	4.3 ± 0.23	4.33 ± 0.23	0.481	4.2 ± 0.2	4.28 ± 0.18	0.065
T-bilirubin (mg/dL)	0.75 ± 0.19	0.75 ± 0.24	0.916	0.86 ± 0.23	0.85 ± 0.22	0.862	0.72 ± 0.18	0.73 ± 0.21	0.950
LD (IU/L)	187.8 ± 37.18	188.3 ± 33.79	0.955	175.35 ± 29.96	179.9 ± 29.13	0.298	174.1 ± 26.8	190.75 ± 31.93	0.001
ALP (IU/L)	60.15 ± 15.66	59.45 ± 12.39	0.673	59.5 ± 11.02	61.3 ± 9.6	0.328	55.75 ± 14.82	58.7 ± 15.24	0.061
CK (IU/L)	93.95 ± 31.32	99.45 ± 38.22	0.496	113.85 ± 54.14	105.95 ± 42.79	0.433	108.2 ± 63.26	138.15 ± 116.88	0.176

Note: WBC, white blood cell; RBC, red blood cell; GGT, gamma-glutamyl transferase; ALT, alanine aminotransferase; AST, aspartate aminotransferase; BUN, blood urea nitrogen; T-protein, total protein; T-bilirubin, total bilirubin; LD, lactate dehydrogenase; ALP, alkaline phosphatase; CK, creatine kinase.

**Table 5:** Effect on metabolic parameters among the three groups.

Value	Group								
	HTC (*n* = 20)			LTC (*n* = 20)			CC (*n* = 20)		
	Before	After	*p*-Value	Before	After	*p*-Value	Before	After	*p*-Value
SBP (mmHG)	129.95 ± 8.51	132.05 ± 11.2	0.430	131.55 ± 10.75	128.15 ± 10.66	0.072	131.35 ± 12.35	131.75 ± 13.15	0.857
DBP (mmHG)	78.15 ± 7.35	77.7 ± 7.95	0.810	79.25 ± 8.28	78 ± 7.31	0.310	78.1 ± 9.24	77 ± 8.28	0.546
Pulse (bpm)	73.6 ± 9.72	78.1 ± 11.38	0.073	72.9 ± 7.7	73.2 ± 6.44	0.810	76.1 ± 8.94	78 ± 8.11	0.191
WC (cm)	88.57 ± 5.51	88.64 ± 5.4	0.312	89.16 ± 7.52	89.12 ± 7.56	0.653	88.01 ± 5.03	87.9 ± 5.07	0.302
HC (cm)	98.34 ± 5.64	98.36 ± 5.58	0.713	98.52 ± 5.48	98.45 ± 5.52	0.368	97.42 ± 4.88	97.48 ± 4.99	0.491
WHR	0.9 ± 0.04	0.9 ± 0.04	0.577	0.91 ± 0.04	0.91 ± 0.04	1.000	0.9 ± 0.04	0.9 ± 0.04	0.258
Weight (kg)	66.59 ± 6.89	66.43 ± 6.64	0.359	65.8 ± 7.46	65.32 ± 8.19	0.246	65.36 ± 7.92	65.17 ± 8.55	0.600
BMI (kg/m^2^)	26.78 ± 2.53	26.72 ± 2.41	0.411	26.48 ± 2.99	26.28 ± 3.31	0.210	26.7 ± 2.55	26.53 ± 2.67	0.209
BFM (kg)	25.06 ± 3.77	24.75 ± 3.85	0.246	24.56 ± 4.28	24.21 ± 4.84	0.233	24.83 ± 4.31	24.41 ± 4.51	0.032
PBF (%)	37.57 ± 3.41	37.17 ± 3.58	0.271	37.24 ± 3.75	36.88 ± 4.22	0.276	37.85 ± 3.3	37.39 ± 3.45	0.030
FFM (kg)	41.53 ± 4.39	41.68 ± 4.26	0.519	41.24 ± 4.78	41.12 ± 4.97	0.593	40.58 ± 4.55	40.62 ± 4.88	0.846
AFR, abdominal fat rate (%)	0.92 ± 0.04	0.92 ± 0.05	0.644	0.91 ± 0.04	0.91 ± 0.04	0.782	0.92 ± 0.04	0.92 ± 0.04	0.700
BMR (kcal/day)	1267 ± 94.69	1270.3 ± 91.88	0.505	1260.8 ± 103.11	1258.1 ± 107.23	0.574	1246.4 ± 98.63	1247.5 ± 105.27	0.827
CRP (mg/L)	2.73 ± 4.92	2.42 ± 3.92	0.475	3.73 ± 6.4	3.33 ± 5.51	0.516	1.73 ± 1.84	2.23 ± 2.23	0.106
ESR (mm/h)	7.12 ± 5.8	5.76 ± 5.58	0.333	6.41 ± 5.93	6.8 ± 7.24	0.653	9.33 ± 8.34	8.73 ± 7.92	0.513
HDL (mg/dL)	51.55 ± 10.37	56.6 ± 15.68	0.068	59.2 ± 15.02	59.2 ± 14.46	1.000	53.7 ± 13.02	55.6 ± 13.57	0.216
LDL (mg/dL)	107.9 ± 36.53	105.15 ± 35.83	0.608	135 ± 54.11	130.8 ± 50	0.531	116.6 ± 27.28	113 ± 33.42	0.408
TC (mg/dL)	193.3 ± 39.28	192.8 ± 36.91	0.939	228.55 ± 53.58	223.15 ± 48.16	0.490	201.45 ± 30.88	200 ± 41.89	0.843
Glucose (mg/dL)	110.4 ± 10.22	104.9 ± 9.29	0.004	110.6 ± 15.45	110.05 ± 18.18	0.826	111.55 ± 16.15	107.8 ± 14.17	0.149
Insulin (µU/mL)	7.1 ± 3.94	7.29 ± 3.58	0.807	6.94 ± 7.1	8.25 ± 6.32	0.386	11.85 ± 13.68	8.45 ± 3.63	0.262
HOMA-IR	1.99 ± 1.2	1.93 ± 1.04	0.764	1.99 ± 2.32	2.35 ± 2.03	0.452	3.55 ± 4.94	2.28 ± 1.07	0.247
HOMA-β (%)	52.58 ± 24.99	62.3 ± 28.36	0.120	50.45 ± 38.32	62.69 ± 37.29	0.186	80.75 ± 62.97	71.61 ± 37.5	0.522
QUICKI	0.36 ± 0.04	0.36 ± 0.03	0.917	0.37 ± 0.05	0.35 ± 0.03	0.104	0.34 ± 0.04	0.35 ± 0.03	0.487

Note: HTC, traditional Cheonggukjang, containing a high dose of beneficial microbes; LTC, traditional Cheonggukjang, containing a low dose of effective microbes; CC, commercially prepared Cheonggukjang; SBP, systolic blood pressure; DBP, diastolic blood pressure; HC, hip circumference; WC, waist circumference; WHR, waist–hip ratio; BMI, body mass index; BFM, body fat mass; PBF, percentage body fat; FFM, fat-free mass; AFR, abdominal fat rate; BMR, basal metabolic rate; hs-CRP, high-sensitivity C-reactive, ESR, erythrocyte sedimentation rate; HDL-C, high-density cholesterol; LDL-C, low-density cholesterol; TC, total cholesterol; HOMA-IR, homeostatic model assessment for insulin resistance; HOMA-β, homeostatic model assessment for β cells; QUICKI, quantitative insulin sensitivity check index.

**Table 6:** Efficacy evaluation of the Kupperman index scores across the three groups.

Value	Group								
	HTC (*n* = 20)			LTC (*n* = 20)			CC (*n* = 20)		
	Before	After	*p*-Value	Before	After	*p*-Value	Before	After	*p*-Value
Vasomotor	8 ± 2.9	6.2 ± 2.04	0.004	8.4 ± 3.65	7 ± 2.2	0.015	8 ± 2.9	7.2 ± 2.78	0.104
Paresthesia	2.8 ± 1.77	2.1 ± 1.37	0.005	2.5 ± 1.57	2 ± 1.3	0.135	3 ± 1.03	2.1 ± 0.45	0.001
Insomnia	4.6 ± 1.47	4.6 ± 1.6	1.000	4 ± 1.45	4.2 ± 1.58	0.494	4.5 ± 1.28	4.6 ± 1.31	0.577
Nervousness	1.3 ± 1.98	0.5 ± 1.1	0.017	1.05 ± 1.36	1 ± 1.21	0.789	1.8 ± 1.82	1 ± 1.21	0.002
Melancholia	0.15 ± 0.49	0 ± 0	0.186	0.2 ± 0.41	0.05 ± 0.22	0.186	0.25 ± 0.55	0 ± 0	0.056
Vertigo	1.95 ± 4.8	0.9 ± 0.85	0.337	1.1 ± 0.97	0.85 ± 0.59	0.135	1.05 ± 0.83	0.75 ± 0.64	0.137
Fatigue	2.7 ± 0.57	2.9 ± 0.31	0.214	2.45 ± 0.89	2.7 ± 0.57	0.204	2.45 ± 0.83	2.75 ± 0.44	0.110
Headache	1.05 ± 0.89	0.85 ± 0.75	0.297	0.9 ± 0.79	0.6 ± 0.6	0.137	0.75 ± 0.64	0.55 ± 0.69	0.104
Arthralgia and myalgia	1.95 ± 1.05	1.85 ± 0.99	0.649	2.15 ± 0.99	1.7 ± 0.86	0.058	2 ± 0.56	2.1 ± 0.79	0.577
Palpitation	0.25 ± 0.64	0.15 ± 0.37	0.330	0.35 ± 0.75	0.15 ± 0.49	0.330	0.1 ± 0.31	0.15 ± 0.37	0.330
Formication	0.05 ± 0.22	0.05 ± 0.22	1.000	0.15 ± 0.49	0.05 ± 0.22	0.428	0.15 ± 0.67	0.05 ± 0.22	0.330
Total	23.8 ± 4.62	20.1 ± 3.24	<0.0001	23.25 ± 4.84	20.25 ± 3.82	0.001	24.15 ± 4.11	21.25 ± 3.18	0.001
Vaginal dryness	1.1 ± 1.02	1.2 ± 0.77	0.494	1.25 ± 1.02	1.2 ± 0.62	0.804	0.85 ± 0.67	1.15 ± 0.67	0.010

Note: HTC, traditional Cheonggukjang containing a high dose of beneficial microbes; LTC, traditional Cheonggukjang containing a low dose of effective microbes; CC, commercially prepared Cheonggukjang.

**Table 7:** Microbiome analysis of feces.

Value	HTC			LTC			CC		
	Before	After	*p*-Value	Before	After	*p*-Value	Before	After	*p*-Value
Firmicutes (%)	71.97 ± 8.03	66.94 ± 10.99	0.106	69.13 ± 10.86	71.66 ± 12.66	0.390	72.75 ± 9.6	70.2 ± 9.69	0.396
Bacteroidetes (%)	15.65 ± 10.44	17.84 ± 10.23	0.491	17.66 ± 10.11	15.76 ± 13.12	0.448	12.88 ± 8.46	16.03 ± 9.1	0.206
F/B	13.01 ± 26.79	10.5 ± 18.83	0.743	12.82 ± 24.05	22.22 ± 34.22	0.232	47.52 ± 127.62	9.07 ± 14.18	0.154
Beneficial bacteria	24.41 ± 7.51	28.41 ± 7.49	0.036	22.82 ± 7.71	23.84 ± 8.96	0.661	27.14 ± 12.37	27.91 ± 11.55	0.725
Harmful bacteria	3.3 ± 3.82	3.9 ± 6.17	0.728	3.1 ± 5.49	2.55 ± 2.39	0.640	4.65 ± 5.72	4.2 ± 4.95	0.544
Others	72.29 ± 7.54	67.69 ± 9.21	0.017	74.08 ± 8.96	73.61 ± 9.06	0.850	68.21 ± 11.51	67.89 ± 11.16	0.892

Note: HTC, traditional Cheonggukjang containing a high dose of beneficial microbes; LTC, traditional Cheonggukjang containing a low dose of effective microbes; CC, commercially prepared Cheonggukjang. Values are presented as mean ± standard deviation or number (percentage).

**Table 8:** Beneficial and harmful microorganisms in the fecal gut microbiome.

Beneficial Microorganism	Beneficial Microorganism	Harmful Microorganism
*Lactobacillus paracasei*	*Lactobacillus delbrueckii*	*Clostridium perfringens*
*Lactobacillus helveticus*	*Bifidobacterium angulatum*	*Bacteroides eggerthii*
*Lactobacillus gasseri*	*Peptostreptococcus anaerobius*	*Sutterella stercoricanis*
*Lactobacillus fermentum*	*Bifidobacterium dentium*	*Ruminococcus torques*
*Lactobacillus plantarum*	*Ruminococcus gnavus*	*Parabacteroides merdae*
*Lactobacillus reuteri*	*flavonifractor plautii*	*Parabacteroides distasonis*
*Lactobacillus salivarius*	*Roseburia inulinivorans*	*Desulfovibrio piger*
*Lactobacillus sakei*	*akkermansia muciniphila*	*Butyrivibrio crossotus*
*Bifidobacterium bifidum*	*Ruminococcus bromil*	*Bacteroides thetaiotaomicron*
*Bifidobacterium breve*	*Closteridium leptum*	*Staphylococcus aureus*
*Bifidobacterium longum*	*Bacillus coagulans*	*Escherichia coli*
*Bifidobacterium animalis*	*Bacillus subtilis*	*Blautia obeum*
*Bifidobacterium adolescentis*	*Bifidobacterium pseudolongum*	*Hafnia alvei*
*Lactococcus lactis*	*Clostridium butyricum*	*Morganella morganii*
*Enterococcus faecium*	*Lactobacillus brevis*	*Bacillus cereus*
*Enterococcus faecalis*	*Lactobacillus delbrueckii*	*Campylobacter jejuni*
*Bacteroides massiliensis*	*Lactococcus lactis subsp. cremoris*	*Streptococcus agalactiae*
*Bacteroides vulgatus*	*Leuconostoc kimchii*	*streptococcus pneumoniae*
*Bacteroides dorei*	*Leuconostoc citreum*	*Acinetobacter lwoffii*
*Alistipes putredinis*	*Leuconostoc mesenteroides*	*Pseudomonas aeruginosa*
*Collinsella intestinalis*	*Pediococcus acidilactici*	*Acinetobacter baumannii*
*Blautia hansenii*	*Streptococcus salivarius*	*Clostridium perfringens*
*Faecalibacterium prausnitzii*	*Weissella confusa*	*Bacteroides eggerthii*
*Odoribacter splanchnicus*	*Bifidobacterium dentium*	*Sutterella stercoricanis*
*Roseburia intestinalis*	*Bacteroides plebeius*	*Ruminococcus torques*
*Collinsella aerofaciens*	*Bacteroides uniformis*	*Parabacteroides merdae*

## Data Availability

The original contributions presented in the study are included in the article; further inquiries can be directed to the corresponding author.
